# Supplementary material for: Perovskite versus ZnCuInS/ZnS Luminescent Nanoparticles in Wavelength-Shifting Layers for Sensor Applications
Source: Sensors (Basel). 2021 May 2;21(9):3165. doi: 10.3390/s21093165 (PMC8124472; doi:10.3390/s21093165)
Supplement: Supplementary file 1 [file sensors-21-03165-s001.zip › sensors-1144315-supplementary.pdf]

Table S1. Composition of all samples.

QDs type	Polymer base	Volume of QDs solution, mL	Volume of polymer base, mL	Volume of toluene added	Percentage of QDs solution to polymer base solution	Polymer to QDs mass ratio	QDs type	Polymer base	Volume of QDs solution, mL	Volume of polymer base, mL	Volume of toluene added	Percentage of QDs solution to polymer base solution	Polymer to QDs mass ratio
PSK 515 0.2 mgl/mL	PMMA 16%	0,1	1,6	1,5	6%	11149	PSK 515 0.1 mgl/mL	Zeonex 15%	0,06	0,6	0,54	10%	13278
		0,1	1,3	1,2	8%	9058			0,12	0,6	0,42	20%	6639
		0,1	1	0,9	10%	6968			0,18	0,6	0,42	30%	4426
		0,1	0,7	0,6	14%	4878			0,24	0,6	0,36	40%	3319
		0,12	0,6	0,48	20%	3484			0,3	0,6	0,3	50%	2656
		0,18	0,6	0,42	30%	2323			0,36	0,6	0,24	60%	2213
		0,24	0,6	0,36	40%	1742			0,42	0,6	0,18	70%	1897
		0,3	0,6	0,3	50%	1394			0,48	0,6	0,12	80%	1660
		0,4	0,6	0,2	67%	1045			0,54	0,6	0,06	90%	1475
		0,5	0,6	0,1	83%	836			0,6	0,6	0	100%	1328
		0,6	0,6	0	100%	697			0,7	0,6	0	120%	1138
PSK 525 0.1 mg/mL	PMMA 10%	0,05	1	0,95	5%	17750	PSK 525 0.1 mg/mL	Zeonex 10%					
		0,1	1	0,9	10%	8875			0,6	0,06	0,54	10%	8775
		0,2	1	0,8	20%	4437			0,6	0,12	0,48	20%	4387
		0,3	1	0,7	30%	2954			0,6	0,18	0,42	30%	2925
		0,4	1	0,6	40%	2215			0,6	0,24	0,36	40%	2194
		0,5	1	0,5	50%	1772			0,6	0,3	0,3	50%	1755
		0,6	1	0,4	60%	1477			0,6	0,4	0,2	60%	1316
		0,7	1	0,3	70%	1266			0,6	0,45	0,15	70%	1170
		0,8	1	0,2	80%	1108			0,6	0,5	0,1	80%	1053
		0,9	1	0,1	90%	985			0,6	0,55	0,05	90%	957
		1	1	0	100%	886			0,6	0,6	0	100%	877
		0,6	0,5	0	120%	740							
ZCIS QDs 2 mg/mL	PMMA 10%	0,06	0,6	0,54	10%	444	ZCIS QDs 2 mg/mL	Zeonex 10%					
		0,12	0,6	0,48	20%	222			0,04	0,6	0,56	7%	656
		0,18	0,6	0,42	30%	148			0,06	0,6	0,54	10%	438
		0,24	0,6	0,36	40%	111			0,16	0,6	0,57	27%	164
		0,3	0,6	0,3	50%	89			0,18	0,6	0,42	30%	146

		0,36	0,6	0,24	60%	74			0,24	0,6	0,36	40%	109
		0,42	0,6	0,18	70%	63			0,36	0,6	0,24	60%	73
		0,48	0,6	0,12	80%	55			0,48	0,6	0,12	80%	55
		0,54	0,6	0,06	90%	49			0,6	0,6	0	100%	44
		0,6	0,6	0	100%	44							
